# Developmental Dynamics of X-Chromosome Dosage Compensation by the DCC and H4K20me1 in *C*. *elegans*

**DOI:** 10.1371/journal.pgen.1005698

**Published:** 2015-12-07

**Authors:** Maxwell Kramer, Anna-Lena Kranz, Amanda Su, Lara H. Winterkorn, Sarah Elizabeth Albritton, Sevinc Ercan

**Affiliations:** Department of Biology, Center for Genomics and Systems Biology, New York University, New York, New York, United States of America; Brown University, UNITED STATES

## Abstract

In *Caenorhabditis elegans*, the dosage compensation complex (DCC) specifically binds to and represses transcription from both X chromosomes in hermaphrodites. The DCC is composed of an X-specific condensin complex that interacts with several proteins. During embryogenesis, DCC starts localizing to the X chromosomes around the 40-cell stage, and is followed by X-enrichment of H4K20me1 between 100-cell to comma stage. Here, we analyzed dosage compensation of the X chromosome between sexes, and the roles of *dpy-27* (condensin subunit), *dpy-21* (non-condensin DCC member), *set-1* (H4K20 monomethylase) and *set-4* (H4K20 di-/tri-methylase) in X chromosome repression using mRNA-seq and ChIP-seq analyses across several developmental time points. We found that the DCC starts repressing the X chromosomes by the 40-cell stage, but X-linked transcript levels remain significantly higher in hermaphrodites compared to males through the comma stage of embryogenesis. *Dpy-27* and *dpy-21* are required for X chromosome repression throughout development, but particularly in early embryos *dpy-27* and *dpy-21* mutations produced distinct expression changes, suggesting a DCC independent role for *dpy-21*. We previously hypothesized that the DCC increases H4K20me1 by reducing *set-4* activity on the X chromosomes. Accordingly, in the *set-4* mutant, H4K20me1 increased more from the autosomes compared to the X, equalizing H4K20me1 level between X and autosomes. H4K20me1 increase on the autosomes led to a slight repression, resulting in a relative effect of X derepression. H4K20me1 depletion in the *set-1* mutant showed greater X derepression compared to equalization of H4K20me1 levels between X and autosomes in the *set-4* mutant, indicating that H4K20me1 level is important, but X to autosomal balance of H4K20me1 contributes only slightly to X-repression. Thus H4K20me1 by itself is not a downstream effector of the DCC. In summary, X chromosome dosage compensation starts in early embryos as the DCC localizes to the X, and is strengthened in later embryogenesis by H4K20me1.

## Author Summary

In many animals, males have a single X and females have two X chromosomes, creating an X chromosomal gene dosage imbalance between sexes. This imbalance is corrected by dosage compensation mechanisms, which are essential for development in mammals, flies and worms. The timing and the molecular mechanisms of dosage compensation during development remain unclear. In *Caenorhabditis elegans*, a complex of proteins called the dosage compensation complex (DCC) binds to and represses transcription of both X chromosomes in hermaphrodites (XX) by half to match X expression to that of males (XO). We found that the DCC starts repressing X chromosomes in early embryogenesis, but average X-linked transcript levels remain higher in hermaphrodite embryos compared to males until the larval stage. Later in embryogenesis, the DCC increases H4K20 monomethylation on the X chromosomes, which is important for dosage compensation. Our results suggest that H4K20 monomethylation does not carry out the transcriptional repressive function by itself but strengthens the DCC’s effect. In summary, the DCC localization starts X chromosome repression in early embryogenesis, and in later embryogenesis sets up a chromatin environment that aids dosage compensation that continues through development.

## Introduction

Dosage compensation equalizes X chromosome gene expression between sexes. Different animals use different strategies of dosage compensation by co-opting diverse mechanisms of gene regulation to the X chromosome [1]. In mammals, dosage compensation transcriptionally inactivates one of the two X chromosomes in XX females to equalize overall X expression to that of XY males. In *Drosophila melanogaster*, the X chromosome is transcribed two-fold higher in XY males. In *Caenorhabditis elegans*, dosage compensation represses both X chromosomes by half in XX hermaphrodites, equalizing overall X-chromosomal transcript levels to that of XO males.

X chromosome dosage compensation is established during, and is essential for development in mammals, *D*. *melanogaster* and *C*. *elegans*. In mice, failure to inactive the X results in continual deterioration of the embryo and death around 10 days post coitum [2–4]. In *D*. *melanogaster*, the dosage compensation complex (male specific lethal (MSL) complex) localizes to the X chromosome at the late blastoderm/early gastrula stage [5,6]. Mutations in any of the four MSL complex members slow development and lead to lethality at the late larval and early pupal stages [7–9]. In *C*. *elegans*, mutations in several dosage compensation complex (DCC) subunits are maternal effect lethal, where the progeny of homozygous null mutant worms die at early larval stages [10–12].

Mammalian X inactivation, *D*. *melanogaster* MSL complex, and the *C*. *elegans* DCC all regulate X chromosome chromatin structure [1,13]. In mammals, X inactivation leads to enrichment of various heterochromatic histone modifications on the inactive X [14,15]. In *D*. *melanogaster*, MSL complex increases H4K16 acetylation on the male X chromosome via its histone acetyl transferase subunit MOF [16,17]. In *C*. *elegans*, DCC binding is required for H4K20me1 enrichment and H4K16ac depletion on X chromosomes in hermaphrodites [18,19]. Although several histone modifications are associated with dosage compensation, it is unclear if these histone modifications act as downstream effectors of the dosage compensation complexes.

In *C*. *elegans*, the five-subunit core of the DCC is a condensin complex, called condensin I^DC^ (hereafter condensin DC) [20]. Condensin DC interacts with additional proteins that have roles in hermaphrodite and X-specific recruitment of the DCC to the X chromosomes [21–24]. Condensins are evolutionarily conserved protein complexes that are essential for chromosome condensation and segregation during cell division (reviewed in [25]). In metazoans, there are two types of condensins, named condensin I and II. In *C*. *elegans*, condensin DC is distinguished from condensin I by a single subunit, DPY-27 [20]. Unlike condensin DC, condensin I and II bind to all chromosomes [20,26]. In *C*. *elegans*, knockdown of condensin II specific subunit KLE-2 suggested that condensin II is also repressive [26]. Condensins have been implicated in transcriptional regulation in other organisms, but the molecular mechanisms by which condensins regulate transcription remain unknown (reviewed in [27]).

**Fig 1:**
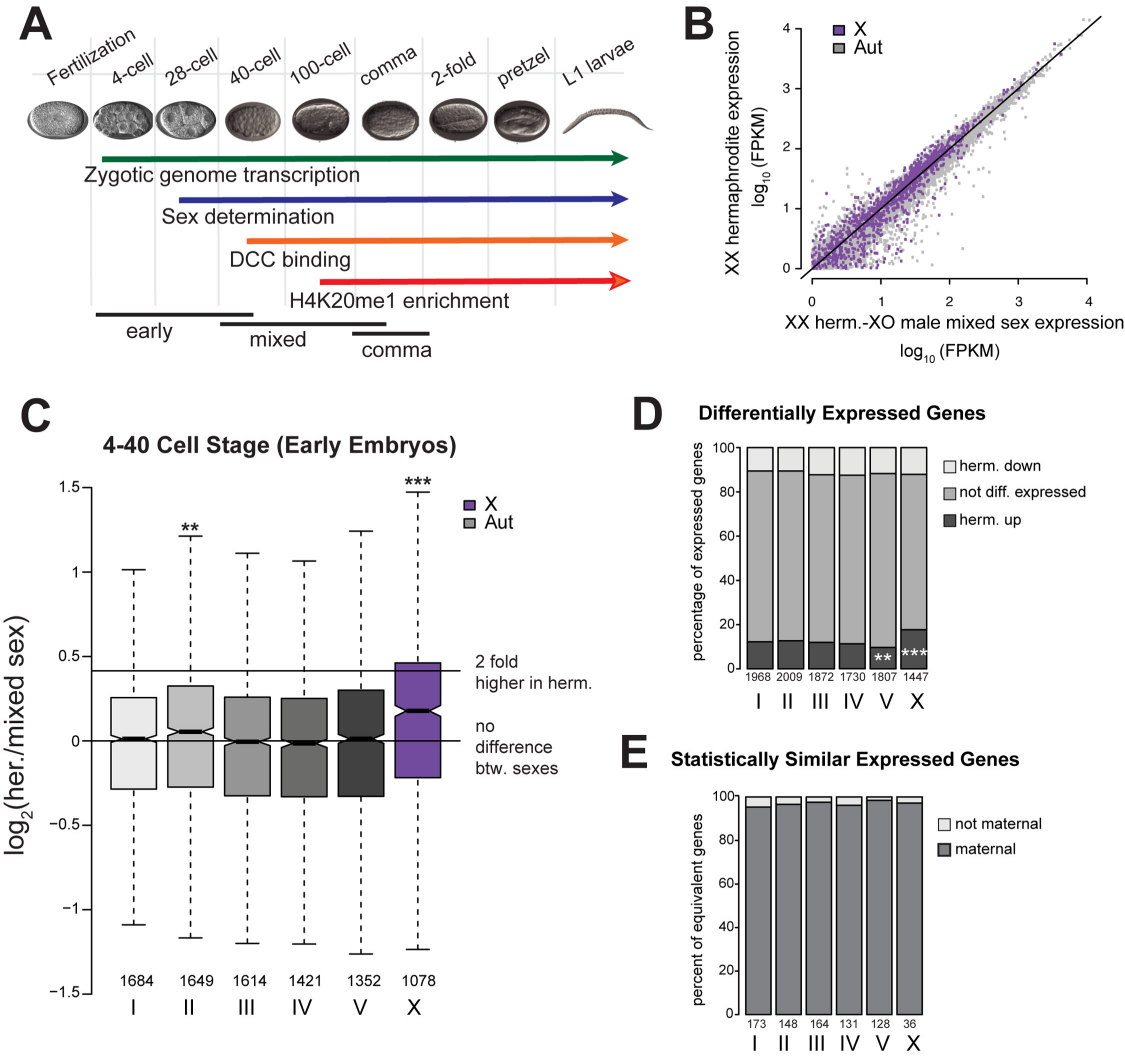
The X chromosome is not fully dosage compensated in early embryogenesis. (A) Timeline of X chromosome regulation during C. elegans embryogenesis. Zygotic transcription begins at the four-cell stage [100,101], followed by expression of sex determination genes by the 28 cell-stage [32]. This triggers DCC localization to the X chromosomes at about the 40-cell stage [21,22,28] and H4K20me1 enrichment on the X chromosome starting at the 100-cell stage [18]. (B) mRNA-seq expression values from hermaphrodites (y-axis) and mixed sex (x-axis) early embryos (4–40 cell stage) were plotted. X is shown in purple, and shifted slightly towards the hermaphrodite data. (C) Boxplot shows distribution of log_2_ fold-difference between hermaphrodites and mixed sex early embryos. Horizontal lines indicate equal expression between sexes (log_2_ fold difference = 0) and 2-fold more expression in hermaphrodites (log_2_ fold difference = 0.415). The n numbers below boxes show the number of expressed genes for each chromosome. Compared to each of the autosomes, X chromosome expression is significantly higher in hermaphrodites (one-sided Wilcoxon rank sum test, *** = p < 10^−3^, ** = p < 10^−2^, and * = p < 0.05.). (D) Differentially expressed genes (DESeq padj < 0.05) were sorted into three groups: up (higher) or downregulated (lower transcript levels) in hermaphrodites, and not differentially expressed. Distribution of these groups among the chromosomes indicated a significant enrichment of hermaphrodite upregulated genes on the X chromosome (Fisher’s exact test for enrichment, *** = p < 10^−3^, ** = p < 10^−2^). (E) Plots indicate the percentage of statistically similarly expressed transcripts (see methods) that were maternally deposited (oocyte FPKM>1 [30]).

*C*. *elegans* DCC is as a clear paradigm for studying the mechanisms of transcription regulation by condensins. The DCC begins localizing to the hermaphrodite X chromosomes at around 40-cell stage (Fig 1A) [22,28]. DCC binding leads to enrichment of H4K20me1 on the X that begins around the 100-cell stage [18,29]. Here, we analyzed X chromosome dosage compensation during *C*. *elegans* development by performing mRNA-seq comparison of gene expression between sexes, and in *dpy-27* (condensin DC subunit) and *dpy-21* (non-condensin DCC member) mutants. Our results suggest that condensin DC starts repression as it localizes to the X chromosomes in early embryos (4–40 cell stage). Gene expression differences between *dpy-21* and *dpy-27* mutants suggest an additional DCC independent role for *dpy-21*, emphasizing that condensin DC core and the non-condensin DCC members functionally differ. To clarify the role of H4K20me1 in X chromosome dosage compensation, we performed mRNA-seq and H4K20me1 ChIP-seq analyses in *set-1* (H4K20 monomethylase) and *set-4* (H4K20 di-/-tri methylase) mutant worms. Our results suggest that H4K20me1 levels are important for X chromosome dosage compensation, but H4K20me1 by itself does not act as a downstream effector of the DCC.

## Results

### X chromosome is not fully dosage compensated during early embryogenesis

We measured X chromosome dosage compensation in early embryos, when zygotic transcription is active but the DCC is not yet fully localized to the X chromosome. Here, we measured dosage compensation of a gene as the ratio of its mRNA levels between hermaphrodites and males. We generated mRNA-seq datasets from 2–5 biological replicates (S1 File) that highly correlated (S1 Fig). Because of the difficulty in obtaining pure male populations prior to the onset of sex specific gene expression, we compared hermaphrodite embryos (XX) to mixed sex (XX and XO) embryos. Mixed sex embryos were isolated by crossing males and hermaphrodites of obligate out-crossing strain (*fog-2(oz40)*) to ensure a 50% male population. “Early embryos” are between 4-cell and 40-cell stages (S2 Fig). At this stage, zygotic transcription is activated and the DCC starts localizing to but not fully enriched on the X chromosome in all cells (Fig 1A) [22,29].

In early embryos, X chromosome expression was higher in hermaphrodites compared to mixed sex (Fig 1B). The median log_2_ ratio of expression between hermaphrodite and mixed sex embryos was significantly higher on the X (0.182) compared to autosomes (ranges from 0.034 to 0.052) (Fig 1C, one sided Wilcoxon rank-sum test p < 1.77 x 10^−9^). Additionally, genes with significantly higher expression in hermaphrodites were enriched on the X (Fig 1D, Fisher’s exact test p = 3.12x10^-6^). Thus, X chromosome is not fully dosage compensated between sexes in early embryos, but X chromosomal transcripts are fairly similar between sexes, as the observed median log_2_ expression ratio of 0.182 is less than expected if X expression were two-fold higher in XX hermaphrodite embryos in the XX/mixed sex comparison (0.411).

### Maternal loading helps equalize X chromosomal gene dosage between sexes in early embryogenesis

One mechanism that might equalize X chromosomal transcript levels between sexes is the maternal loading of mRNAs into oocytes. To test this, we asked if similarly expressed genes are maternally loaded. While most methods identify genes that are differentially expressed between sexes, a lack of differential expression does not show that genes are *statistically* similarly expressed. We identified 780 genes that are statistically similarly expressed between hermaphrodite and mixed sex early embryos by taking genes that show less than 30% difference in expression within a 95% confidence interval (see Materials and Methods for full explanation). Among the 36 similarly expressed X-chromosomal genes, 35 were maternally loaded (FPKM>1 in oocytes [30]). Approximately 95% of similarly expressed genes on autosomes were also maternally loaded, thus maternal loading of transcripts into oocytes helps equalize gene dosage between hermaphrodite and male embryos (Fig 1E).

### Dosage compensation of zygotic gene expression during early embryogenesis

**Fig 2:**
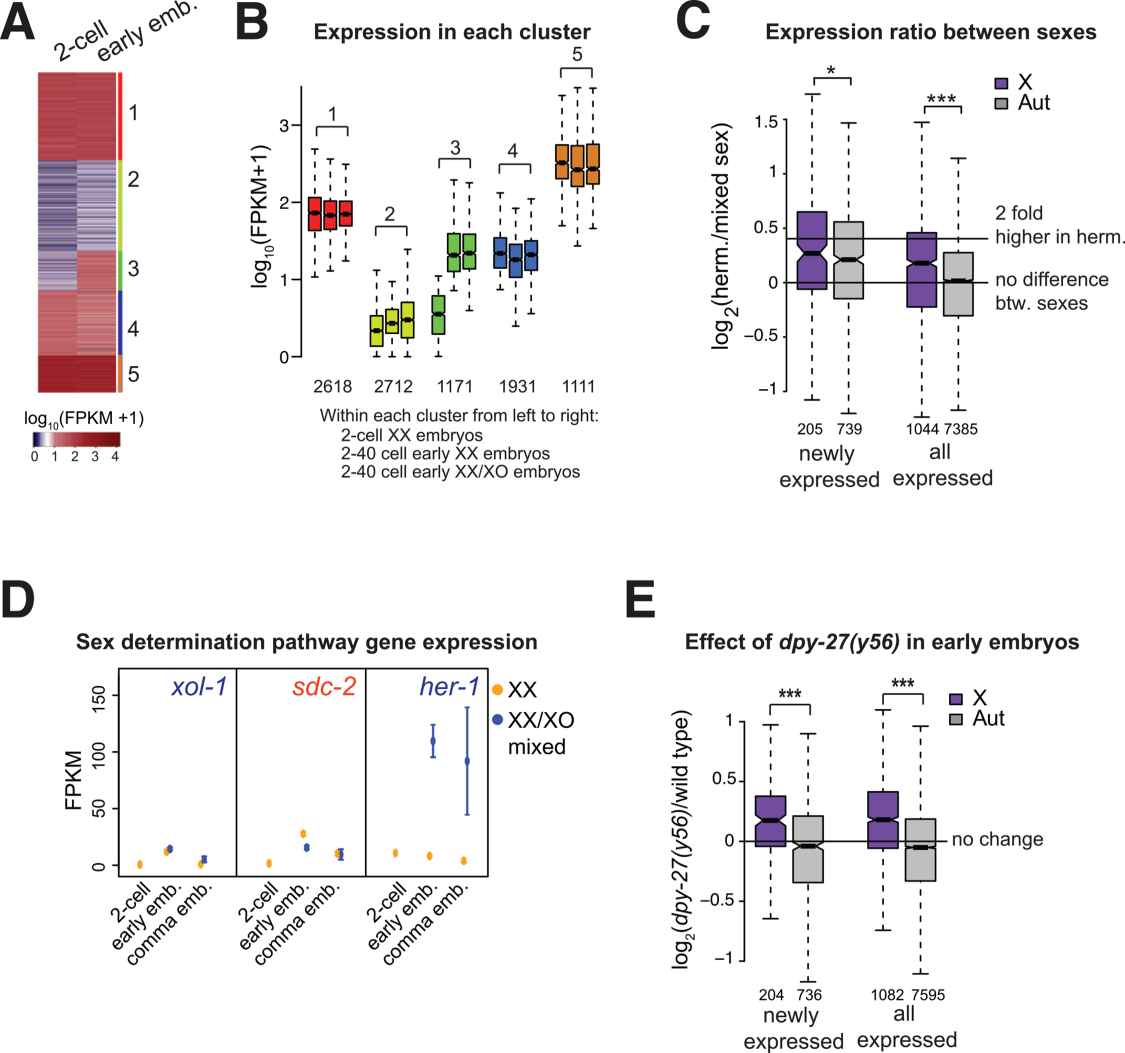
DCC represses zygotic expression of the X chromosome in early embryos. (A) K means clustering of expression in 2-cell [30] and early hermaphrodite embryos (4–40 cell). Of the five clusters identified, one showed clearly increased expression between 2-cell and early embryos (#3). (B) Boxplots show expression levels for genes separated by clusters in 2-cell [30] and early hermaphrodite and mixed sex embryos. (C) Newly expressed genes were identified by taking genes in cluster #3 that showed no expression in 2-cell embryos (FPKM<1). Boxplots show distribution of expression difference between sexes in early embryos. Both newly expressed, and all expressed (FPKM>1) genes were significantly higher in hermaphrodite X chromosomes compared to autosomes (one sided Wilcoxon rank-sum test, *** = p < 10^−3^, * = p < 0.05) (D) Expression level of early sex determination genes in hermaphrodites and mixed sex samples are shown for the indicated data sets. 2-cell stage expression data in hermaphrodite embryos were obtained from [30]. Error bars show the standard error of the mean. (E) Log_2_ expression ratios between *dpy-27(y56)* and N2 wild type show significant X derepression in *dpy-27(y56)* early embryos. *dpy-27(y56)* mutation affects expression of newly expressed zygotic genes (panel C) on the X chromosome compared to autosomes. Significance of X upregulation was tested using one sided Wilcoxon rank-sum test (*** = p < 10^−3^).

To specifically study dosage compensation of zygotic gene expression, we identified genes that are not expressed in 2-cell stage embryos but are newly expressed in early embryos. K-means clustering of expression between previously published expression data in 2-cell hermaphrodite embryos [30] and our data in early embryos resulted in five distinct clusters (Fig 2A). Genes in cluster 3 are zygotic because they showed little or no expression in 2-cell embryos and increased expression in early embryos (Fig 2B). Since we cannot distinguish between stable and newly transcribed mRNAs, genes in the remaining clusters may also be zygotically expressed. Thus, to study zygotic expression, we focused on the newly expressed genes (cluster 3). Hermaphrodite expression from the X chromosome was significantly higher compared to the autosomes (one sided Wilcoxon rank sum test, p = 0.019), but the difference was less than expected if there were no dosage compensation. Therefore, early zygotic expression from the X is dosage compensated, but not completely (Fig 2C).

### Expression of sex determination genes in early embryogenesis

In *C*. *elegans*, incomplete dosage compensation in early embryos is logical, as sex is determined by the ratio of X and autosomal sex elements (XSEs and ASEs) [31–33]. In early embryos, we observed that the male-specific *her-1* gene is expressed 11.2 fold higher in mixed-sex samples compared to hermaphrodites (Fig 2D). This corresponds to an estimated 17 fold higher expression in males, consistent with previous measurements of ~20-fold difference in *her-1* expression between males and hermaphrodites [34]. Interestingly, male promoting gene *xol-1* transcript was only slightly higher in mixed sex samples (Fig 2D). It is possible that the difference in endogenous *xol-1* transcript level between sexes is smaller than seen for transgenes [35]. It is also possible that the X-linked *xol-1* is being repressed by the DCC in early embryos, as *dpy-27* starts to repress the X chromosomes in early embryos (Fig 2E), and *xol-1* was shown to be repressed by the DCC [36]. We also found that mRNA levels of both XSEs and ASEs were higher in hermaphrodites, suggesting that XSE and ASE dosage does not function through a simple ratio of mRNA levels between sexes (S3 Fig). Instead, small and transient differences in the transcription of XSEs, ASEs and *xol-1* may be amplified post-transcriptionally to ultimately regulate *her-1* expression, which is clearly higher in males (Fig 2D).

### DPY-27 represses hermaphrodite X chromosomes in early embryogenesis

The DCC is coupled to sex determination through *sdc-2*, which is specifically expressed in hermaphrodite embryos [22]. SDC-2 is required for DCC localization to the X, and for repressing male-specific *her-1* gene [37]. A recent study showed that in 50-cell stage embryos, half the cells contain DPY-27 specifically on the X chromosomes [29]. In our early embryo collections (4–40 cell stage), *sdc-2* is more highly expressed in hermaphrodites and *her-1* is expressed in males, therefore the DCC is activated (Fig 2D). To test if the DCC represses X-chromosome expression shortly after localizing to the X, we collected *dpy-27* null mutant embryos using a strain with genetically balanced *(y56)* allele (Materials and Methods). In early embryos, *dpy-27* null mutation caused significant derepression of newly transcribed zygotic genes on the X (Fig 2E), suggesting that *dpy-27* represses X chromosomes in early embryos. *Dpy-27* is not detectable in the XX hermaphrodite germline by immunostaining, but it is maternally deposited and required for development [38–40]. Therefore, we interpret the observed *dpy-27* null mutation effect coming from the embryos that loaded the DCC onto the X chromosomes in some cells by the 40-cell stage. This implies that the DCC is able to repress X chromosomes quickly after loading.

### X chromosome dosage compensation during development

**Fig 3:**
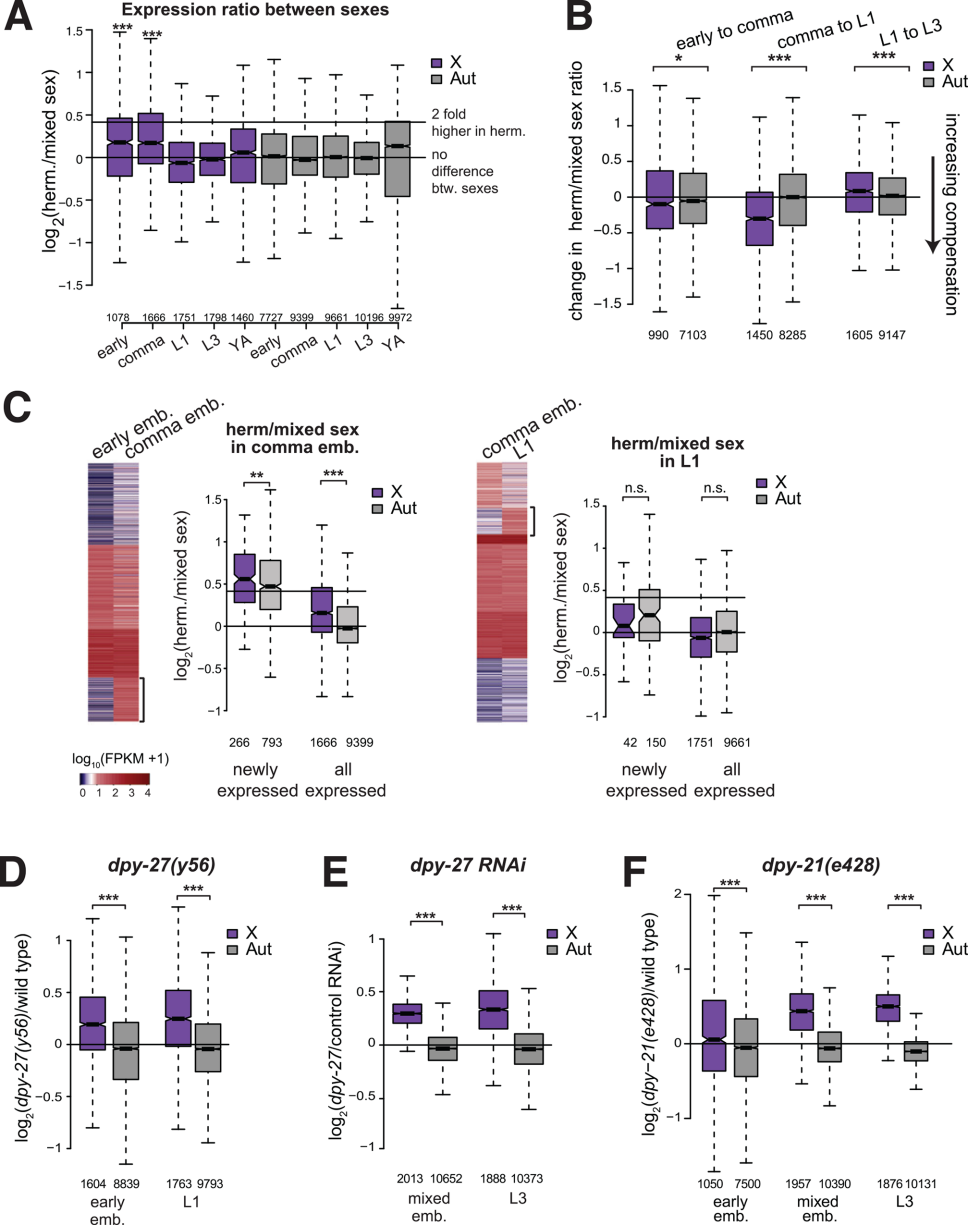
Majority of X chromosomal genes are dosage compensated after the comma stage of embryogenesis. (A) Boxplot shows the distribution of log_2_ fold-differences in mRNA levels between hermaphrodite and mixed sex worms for early embryos, comma stage embryos, L1 larvae, L3 larvae and young adults. Horizontal lines indicate ratio expected for equal and two-fold higher expression in hermaphrodites. Increased expression from the X was tested between X and autosomes by one-sided Wilcoxon rank-sum test (*** = p < 10^−3^). (B) Change in dosage compensation was calculated for each gene by subtracting log_2_(herm/mixed sex) at younger stage from the older stage. Difference in between X and autosome was tested by Wilcoxon rank-sum test (*** = p < 10^−3^, * = p < 0.05). (C) To identify newly expressed genes in comma embryos and L1 larvae, heat maps show K-means cluster analysis of expression levels in hermaphrodites between stages. Newly expressed genes in early embryos were identified as in Fig 2C. In comma embryos, hermaphrodite levels of newly expressed genes was significantly higher for X chromosomal genes compared to autosomal genes (one-sided Wilcoxon rank sum test, *** = p < 10^−3^, ** = p < 10^−2^, and * = p < 0.05.). In L1s, hermaphrodite to mixed sex ratio of expression was not significantly different between the X and autosomes, suggesting that these genes were mostly dosage compensated. (D) Boxplots show distribution of expression ratios on the X and autosomes between *dpy-27(y56)* mutant and wild type, and (E) between *dpy-27* and control RNAi in the developmental stages listed below. X chromosome was significantly derepressed compared to autosomes in *dpy-27(y56)* mutant and *dpy-27* RNAi at each developmental stage (one-sided Wilcoxon rank-sum test *** = p < 10^−3^). (F) Same as in (D) for the *dpy-21(e428)* mutant.

To determine if and when average X chromosome expression is equalized between sexes, we analyzed further time points including comma stage embryos, L1 and L3 larval stages, and young adults. At the comma stage, X chromosome expression remained slightly but significantly higher in hermaphrodites compared to mixed sex worms (Fig 3A). In, L1, L3 larvae and young adults, average X-chromosome expression was no longer higher in hermaphrodites. In addition to measuring dosage compensation at each time point, for each gene we also calculated change in dosage compensation between two consecutive time points. From early to comma stage embryos, the X chromosome was slightly repressed, but X chromosome gene expression became significantly more balanced between sexes after the comma stage (Fig 3B, Wilcoxon rank sum p = 1.13 x 10^−63^). To address if more genes are dosage compensated after the comma stage, we identified the statistically similarly expressed genes at each developmental stage. The percentage of X chromosomal genes that were statistically similarly expressed between sexes increased from early to comma stage embryos (4.6% to 12.8%, respectively), and stayed at similar levels in L1, L3 and young adults (10.3%, 10.4%, 13.5%, respectively). Thus, on average more X chromosomal genes are repressed after the comma stage (Fig 3B), but the proportion of equivalently expressed X chromosomal genes increases after early embryogenesis.

In comma stage embryos, average X expression may be higher in hermaphrodites due to stable transcripts retained from early embryos. To test this, we used K means cluster analysis and further filtering to identify those genes that are newly expressed in comma stage embryos and in L1 larvae (Fig 3C). The newly expressed X chromosomal genes in L1 larvae were dosage compensated and repressed by the DCC (Figs 3C and S5). In comma stage embryos, the newly expressed X chromosomal genes showed significantly higher hermaphrodite expression compared to autosomal genes, thus transcript stability does not fully explain significant hermaphrodite-biased expression from the X chromosomes. It is possible that in comma embryos, X is expressed higher in hermaphrodites because DCC-mediated dosage compensation is incomplete or more genes on the X chromosome have hermaphrodite-biased expression.

### *dpy-27* is required for X chromosome repression throughout development

Comparison of gene expression between sexes is a measure of dosage compensation, but sex-biased gene expression confounds the interpretation of the data (see discussion). To specifically study DCC mediated X repression, we analyzed gene expression changes in hermaphrodites mutant for *dpy-27* or upon *dpy-27* RNAi knockdown. As mutants and RNAi treated worms showed more variability in staging, we mainly used “mixed stage embryos” isolated by bleaching gravid adults. Mixed stage embryos contained 100–300 cells (S4A Fig). We used *dpy-27* RNAi in mixed embryos and L3, because of the difficulty collecting *dpy-27(y56)* null mutant. Western blot analyses showed ~70% and ~40% knockdown of DPY-27 in embryos and L3s, respectively (S4B Fig). Mutation (Fig 3D) or depletion (Fig 3E) of *dpy-27* caused significant X chromosome derepression in early and mixed-stage embryos, L1 and L3 worms. Analysis of newly expressed genes in each stage also indicated that condensin DC represses X chromosomes throughout these developmental stages (S5 Fig). Therefore, although previous studies that used temperature sensitive mutants of various DCC subunits found that the critical time period for DCC activity is centered around the comma stage of embryogenesis, our results suggest that the DCC acts throughout development [12].

### Differences in gene regulation by condensin DC subunit *dpy-27* and non-condensin member of the DCC *dpy-21*

We also analyzed *dpy-21*, a non-condensin subunit of the DCC, whose null mutation (*e428*) is not lethal, but leads to X chromosome dosage compensation defects including a dumpy phenotype [10,11,41]. In mixed embryos and L3, *dpy-21(e428)* mutation also caused X chromosome derepression (Fig 3F). In early embryos, *dpy-21(e428)* had a relatively less X-specific effect compared to *dpy-27(y56)*. In *dpy-21(e428)* early embryos, 72% of X and 58% of autosomal genes were significantly upregulated. In *dpy-27(y56)* early embryos, DESeq analysis did not identify any genes that met the significance cutoff (adjusted p value < 0.05). But, among the top five percent of *dpy-27* regulated genes, 81% of X and 31% autosomal genes were upregulated. The effect of *dpy-21(e428)* on X chromosome expression was more specific in the later stage worms. Clustering and heatmap analysis of gene expression changes showed that *dpy-21(e428)* caused both increased and decreased expression from the X chromosomes in early embryos, whereas majority of the X chromosomal genes were derepressed in comma stage embryos and L3s (S4C Fig). A recent study noted that regulation of H4K16 acetylation on the X chromosomes differs between *dpy-21* and other DCC subunits in early embryos [29]. Since regulation of X chromosome expression also differed between *dpy-27(y56)* and *dpy-21(e428)* mutants, *dpy-21* may have a DCC-independent role in early embryos.

### The DCC represses transcription continuously across the X chromosome

**Fig 4:**
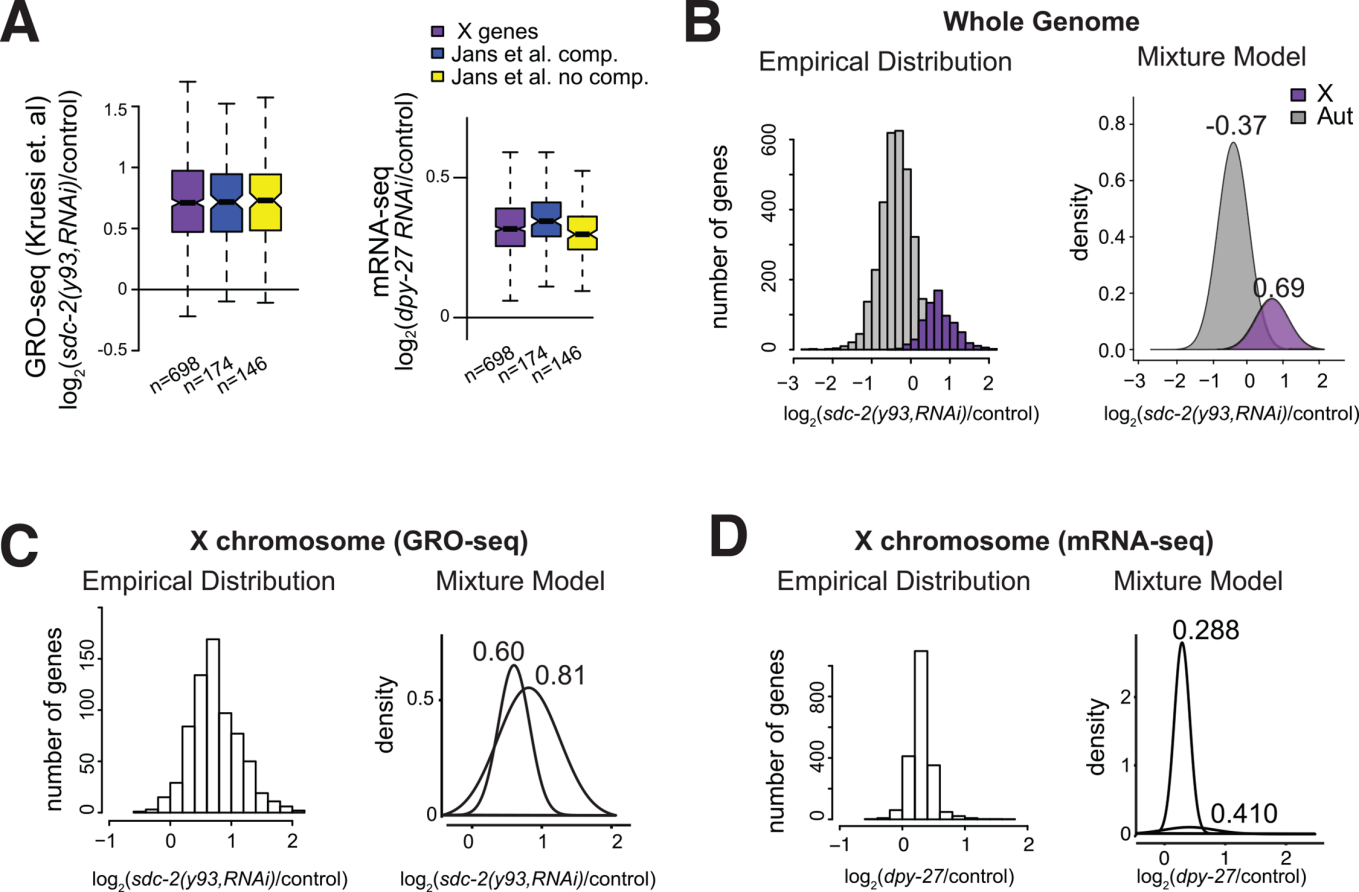
DCC-mediated transcriptional repression is applied continuously across the X chromosomal genes. (A) Box plots show the distribution of GRO-seq gene-body log_2_ ratios in *sdc-2(y93*,*RNAi)* versus control mixed stage embryos taken from published data [43]. Dosage compensated (blue) and non-compensated (yellow) genes were previously categorized by microarray experiments [42]. On average, previously designated compensated and noncompensated classes showed similar upregulation of transcription upon *sdc-2(y93*,*RNAi)* treatment. Same analysis was performed using mRNA-seq data comparing *dpy-27* versus control RNAi in mixed embryos. In this case, noncompensated classes showed less upregulation measured by mRNA-seq. (B) A mixture model was used to represent the presence of subpopulations within an overall population, and applied to the GRO-seq expression ratios of all measured genes. This model identified two distributions similar to the empirical X and autosomal distributions. Values above modeled distributions show the mean for each distribution. (C, D) Same mixture model approach was used to analyze subpopulations within only the X chromosome using the GRO-seq (C), and *dpy-27(y56)* mRNA-seq (D) data in mixed embryos. Both subpopulations showed upregulation, thus the effect of the DCC appears continuous such that no large distinct class of genes completely escapes DCC regulation.

An earlier microarray study suggested that approximately half of X-chromosomal genes are dosage compensated. These genes were identified by the criteria that they were upregulated in the DCC mutants and were equally expressed between XX and XO hermaphrodite embryos [42]. Subsequently, global run on analysis (GRO-seq) of active transcription in *sdc-2(y93*,*RNAi)* embryos showed a uniform increase in RNA Pol II levels across most X chromosomal genes [43], suggesting that there is no large distinct set of genes that escape from DCC-mediated repression. In agreement with a continuous effect, our analysis of the published GRO-seq data showed similar upregulation between previously categorized dosage compensated and non-compensated genes (Fig 4A). In mixed stage embryos, *dpy-27* RNAi mRNA-seq also showed derepression of X chromosomal genes. Derepression was slightly stronger for previously defined compensated genes, suggesting that posttranscriptional regulation contributes to the final mRNA levels in the DCC mutants.

To further test if the DCC acts on a distinct set of genes on the X, we analyzed the distribution of expression changes caused by DCC disruption. We used a standard expectation-maximization algorithm that mixes multiple normal distributions to model the overall pattern of expression changes. We reasoned that if there is a large group of genes specifically repressed by the DCC, when two distributions are forced on the data, one distribution should reflect expression of DCC regulated genes and another should reflect genes not regulated by the DCC. Indeed, when applied to the whole genome GRO-seq data, two distributions can be modeled. Separation of two distributions with average fold changes of -0.37 and 0.69 closely mirrored the distributions of changes for the autosomes and X chromosome (Fig 4B). This indicated that the analysis is sensitive to finding differentially regulated genes by the DCC. Next, we applied the same method to the X chromosome, using GRO-seq data (Fig 4C), and the mRNA-seq data from *dpy-27(y56)* L1s (Fig 4D). In both cases, the separated distributions all showed upregulation, suggesting that there are no large group of genes that escape DCC regulation on the X. This does not exclude the possibility that different groups of genes are affected at different levels, but this effect must be continuous, rather than discrete.

### Mutations in *set-1* and *set-4* affect X chromosome dosage compensation

**Fig 5:**
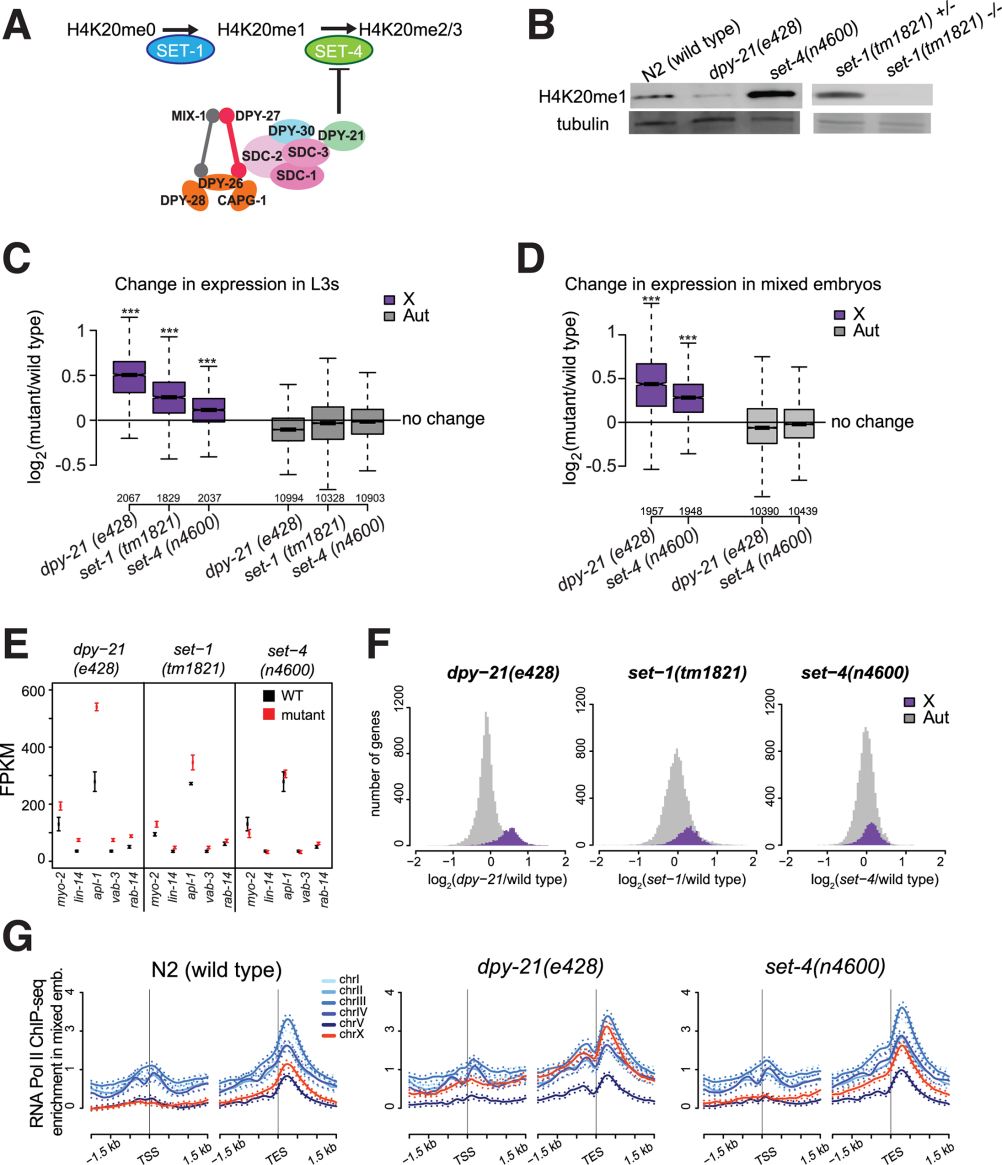
Proper regulation of H4K20 methylation is important for X chromosome dosage compensation. (A) H4K20 methylation is catalyzed in two steps by the histone methyl transferases SET-1 and SET-4. Previous work suggested that the DCC causes H4K20me1 enrichment on the X chromosome by reducing SET-4 catalyzed conversion of H4K20me1 to H4K20me2/3 on the X [18]. (B) Western blot analysis of H4K20me1 levels in wild type N2, *set-4(n4600)*, *dpy-21(e428)*, and in set-1*(tm1821)* null mutant L3 larvae. (C, D) Distribution of log_2_ fold-differences in mRNA levels between mutant and wild type worms in (C) L3 larvae and (D)mixed embryos. All three mutations showed a significant difference between X and autosomal expression changes (Wilcoxon rank-sum test, *** = p < 10^−3^). (E) L3 mRNA-seq differential expression values are plotted for the previously designated dosage compensated genes [11,42,102]. Error bars show the standard error of the mean. (F) The distribution of expression changes (x-axis) in the three mutants show a shift in X chromosome expression, thus the difference between X and autosomes in panel C is not driven by a small number of genes. (G) Average RNA Polymerase II (AMA-1) ChIP-seq enrichment for each chromosome is plotted across the transcription start (TSS) and transcription end sites (TES) in wild type, *dpy-21(e428) and set-4(n4600)* mixed stage embryos.

In *C*. *elegans*, H4K20 is monomethylated by SET-1, and H4K20me1 is converted to di- and tri-methylation by SET-4 [18,19]. Concurrent enrichment of H4K20me1 and depletion of H4K20me3 on the X chromosomes suggested that the DCC increases H4K20me1 levels on the X by reducing SET-4 activity [18] (Fig 5A). X-enrichment of H4K20me1 requires the DCC subunits, including *dpy-21* [18]. We analyzed genome-wide expression changes that occur in *dpy-21(e428)*, *set-1(tm1821)* and *set-4(n4600)* null mutants [18,19]. *Set-1* null mutant is maternal effect sterile, and RNAi knockdown of *set-1* leads to germline deficient adults, thus we could not isolate embryos. Therefore, we collected *set-1(tm1821)* homozygous and heterozygous L3 larvae (see Material and Methods). Western blot analysis in whole-larval extracts showed expected changes in H4K20me1 (Fig 5B) [18,19]. H4K20me1 reduced in *dpy-21(e428)*, increased in *set-4(n4600)*, and was eliminated in *set-1(tm1821)*. Immunofluorescence analysis of intestinal nuclei had also shown that H4K20me1 is higher on X in N2 wild type worms; decreases to the autosomal levels in *dpy-21(e428)* mutant; and increases and becomes equal between X and autosomes in *set-4(n4600)* mutant [19].

H4K20me1 reduction due to *set-1* or *dpy-21* null mutation led to a significant increase in X chromosome expression compared to autosomes in L3 larvae (Fig 5C). We observed a small but significant increase in X expression compared to autosomes in the *set-4(n4600)* mutant L3 (Fig 5C) and mixed stage embryos (Fig 5D). Expression of previously defined dosage compensated genes [11,23,42] were increased in *dpy-21(e428)*, *set-1(tm1821)*, but not as much in *set-4(n4600)* mutant (Fig 5E). To test if a subset of X chromosomal genes were responsible for the effect seen in the *set-4(n4600)* mutant (Fig 5C), we plotted the distribution of expression ratios on the X and autosomes (Fig 5F). This indicated a slight shift between X and autosomes, suggesting that *set-4(n4600)* has a subtle effect across all genes, rather than a large effect on a subset of X chromosomal genes. Note that mRNA-seq measures relative expression changes, rather than absolute. Therefore X derepression seen in the *set-4(n4600)* mutant may not be specific derepression of the X, as discussed later in the results. Nevertheless, the effects of *set-1* and *set-4* mutations indicate that proper regulation of H4K20 methylation is important for X chromosome dosage compensation.

### *dpy-21* and *set-4* regulate RNA Pol II binding

GRO-seq analysis of *sdc-2(y93*, *RNAi)* embryos showed that the DCC reduces RNA Pol II at the X chromosome promoters [43]. To test if the expression changes seen in *dpy-21(e428)* and *set-4(n4600)* mutants are due to transcription, we performed AMA-1 (RNA Pol II large subunit) ChIP-seq analysis. Average RNA Pol II enrichment across the transcription start and end sites showed a 3’ accumulation that was also noted in the GRO-seq analysis for *C*. *elegans* genes (Fig 5G) [43]. RNA Pol II on the X chromosome promoters increased relative to autosomes in *dpy-21(e428)* mutant (Fig 5G). In the *set-4(n4600)* mutant (Fig 5G), there was a subtle shift in RNA Pol II levels between the X and autosomes, suggesting that the effect of *set-4* is also at the level of transcription. These results do not exclude post-transcriptional effects contributing to increased X expression in the *dpy-21(e428)* and *set-4(n4600)* mutants, but do suggest that *dpy-21* and *set-4* act at the level of transcription.

### Differential expression in *dpy-27* RNAi, *dpy-21*, *set-1* and *set-4* mutant L3s

**Fig 6:**
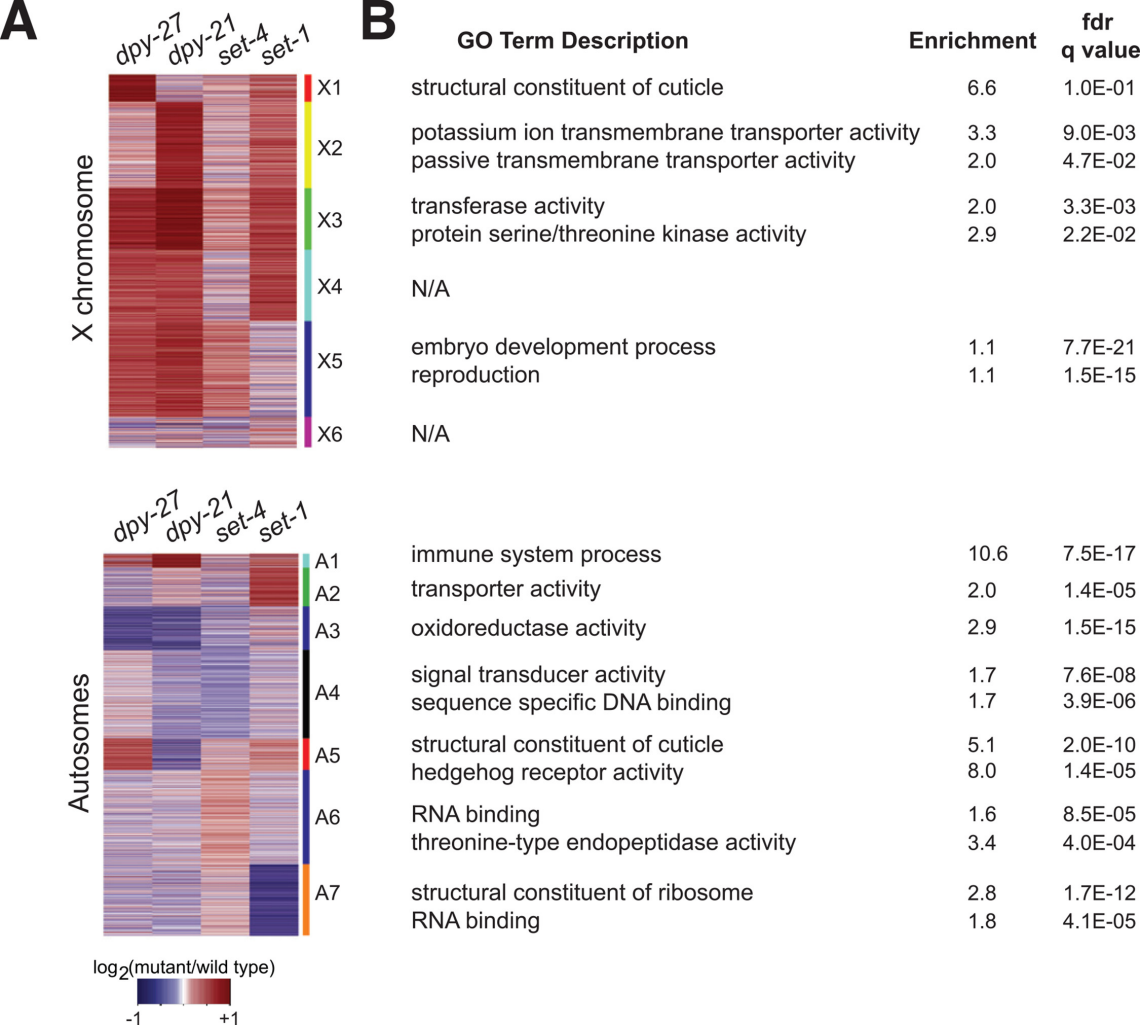
Gene regulation by *dpy-27*, *dpy-21*, *set-4* and *set-1* across the X chromosome and autosomes. (A) K-means clustering was performed using log_2_ ratios of expression in *dpy-27*/control RNAi, *dpy-21(e428)*/N2, *set-4(n4600)*/N2, *set-1(tm1821)* homozygous/heterozygous L3 worms. The clusters are indicated with a color and number to the right of the heat map. (B) GO term analyses indicate the enrichment of gene functions for each cluster compared to the rest of the X or autosomes. Additional GO terms are provided in S4 File.

Unlike other DCC subunits, *dpy-21* is not essential, but is required for H4K20me1 enrichment on the X chromosomes [11,18,19]. To understand the role of *dpy-21* in the DCC, we compared gene expression changes in *dpy-27* RNAi, *dpy-21(e428)*, *set-1(tm1821)* and *set-4(n4600)* mutant L3 larvae by clustering differential expression ratios on the X chromosome and autosomes (Fig 6A). We note that while the effect of *dpy-27* RNAi and *dpy-21(e428)* were generally similar on the X, genes in several clusters were affected differently between *dpy-27* RNAi and *dpy-21(e428)*. We performed gene ontology (GO) analyses comparing enriched functions in each cluster compared to the whole genome (Fig 6B). Interestingly, the effect of *dpy-27* RNAi and *dpy-21(e428)* on clusters enriched for cuticle genes were opposite, yet both mutations result in a dumpy phenotype.

### H4K20me1 increases genome-wide in the *set-4* mutant, equalizing X and autosomal levels

**Fig 7:**
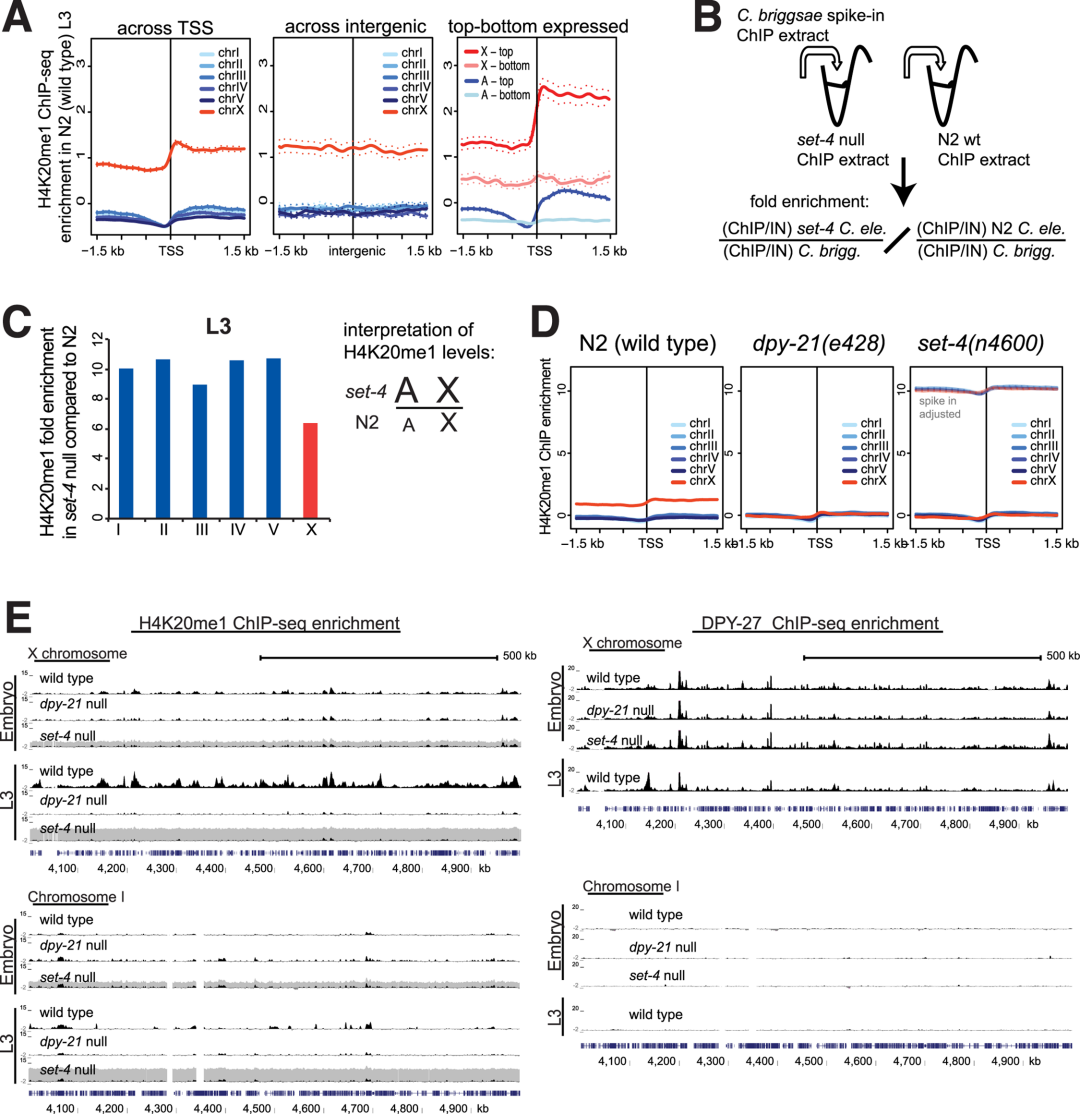
Spike-in normalized analyses of H4K20me1 in *set-4(n4600)* null mutant suggests a genome-wide increase that equalizes X and autosomal levels of H4K20me1. (A) H4K20me1 ChIP-seq in N2 wild type L3 worms. Average ChIP-seq enrichment scores were plotted for each chromosome across transcription start sites (TSS), across middle of intergenic regions that are >5 kb away from any gene, and across TSSs of the upper and lower quintile of expressed genes. (B) Schematic representation of the spike-in ChIP-seq experiment. ChIP extract prepared from *C*. *briggsae* were mixed with extracts prepared from *C*. *elegans* worms prior to H4K20me1 ChIP. The spiked in *C*. *briggsae* extract is the same across wild type and mutant samples. H4K20me1 ChIP enrichment based on total reads that map uniquely to *C*. *elegans* was normalized by the enrichment calculated from reads that map uniquely to the *C*. *briggsae* genome. For each data set, input sample was also sequenced, providing an internal control. (C) Spike in corrected H4K20me1 enrichment ratios reflect the level of H4K20me1 increase in *set-4(n4600)* mutants compared to wild type for each chromosome in L3 worms. (D) Average H4K20me1 ChIP-seq enrichment was plotted across the transcription start sites in wild type, *dpy-21(e428)* and *set-4(n4600)* L3 larvae. Dark lines are from traditionally normalized data. The y-axis scale was increased compared to panel A, to accommodate for inferred enrichment (lighter colored lines) in *set-4(n4600)* based on the spike-in correction ratios from panel C. (E) Representative UCSC genome browser views of ChIP-seq scores for H4K20me1 (left panel) and DPY-27 (right panel). For *set-4(n4600)*, inferred enrichment based on spike-in shifted scores was plotted in grey behind the traditionally normalized ChIP-seq ratios.

Since our model for DCC regulation of H4K20me1 posits that the DCC reduces SET-4 activity, X derepression in the *set-4* mutant was somewhat unexpected. To study this further, we performed H4K20me1 ChIP-seq analyses in wild type, *dpy-21(e428)* and *set-4(n4600)* mutant embryos and L3 larvae. Experimental replicates correlated well with each other (S6A Fig). H4K20me1 is enriched in active gene bodies in multiple species [44]. In *C*. *elegans* hermaphrodite L3s, H4K20me1 is enriched on expressed genes, but also across the entire X chromosome including silent genes and intergenic regions (Fig 7A). Such pattern of H4K20me1 enrichment on the X could be achieved by a uniform deposition of H4K20me1 by SET-1 during M phase, followed by DCC-mediated reduction of SET-4 activity across the X chromosome [18]. However, standard ChIP-seq analysis cannot detect a uniform increase, due to the analysis step that normalizes read depth between experiments [45,46]. Therefore, to analyze change in H4K20me1 in the absence of SET-4, we used *C*. *briggsae* ChIP extract as a spike-in control, similar to previous approaches (Fig 7B) [45,47]. The replicates correlated with each other (S6B Fig). Here, we assume that the ChIP-seq enrichment calculated from the *C*. *briggsae* reads should be the same between *set-4(n4600)* and wild type, since the spiked in *C*. *briggsae* extract is the same. For each ChIP sample, the corresponding input was also sequenced, thus the normalization is internally controlled against any potential difference in the spiked in proportion. We normalized H4K20me1 ChIP enrichment from *C*. *elegans* reads to that of *C*. *briggsae* reads, and calculated the ratio of H4K20me1 levels between *set-4(n4600)* and wild type worms (Fig 7B).

In *set-4(n4600)* L3s, there was ~10-fold increase in H4K20me1 on autosomes and ~6 fold increase on the X chromosome (Fig 7C). This is consistent with the idea that in the wild type worms, the DCC already reduces SET-4 activity on the X by half, thus in the absence of SET-4, H4K20me1 increase on autosomes is approximately two-fold more compared to the increase on the X. Immunofluorescence studies in *set-4* null mutants also showed an overall H4K20me1 increase resulting in the equalization of X and autosomal H4K20me1 levels [18,19]. Plotting H4K20me1 enrichment as measured by standard ChIP-seq analysis across transcription start sites illustrates the equalization of H4K20me1 levels between X and autosomes in *dpy-21(e428)* and *set-4(n4600)* mutants (Figs 7D and S7A). In these plots, ChIP-seq experiments were performed without the spike-in control, and the genome-wide increase of H4K20me1 in the *set-4(n4600)* mutant was masked. Adjusting enrichment values by the spike-in ratios obtained from Fig 7C illustrates the inferred H4K20me1 levels in the *set-4(n4600)* mutant L3s (lighter colors in Fig 7D). The ten-fold increase in H4K20me1 levels across the genome was surprising, based on the observation that western blot analysis of H4K20me1 showed approximately five-fold increase in the *set-4* null mutant L3s (Fig 4B). The basis of this difference between the two different assays (ChIP versus western blot) is unclear. A ten-fold increase of H4K20me1 may be possible in the light of previous studies in mammalian cells, which showed that ~80% of H4 is di-methylated at H4K20 [48,49]. If demethylation of H4K20me1 is inefficient in *C*. *elegans*, *set-4* mutation may result in high levels of monomethylation.

### Delay in H4K20me1 enrichment and dosage compensation

The magnitude of H4K20me1 increase and the difference between X and autosomes was greater in L3s compared to mixed stage embryos in *set-4(n4600)* mutant (S7B Fig). This may be due to less H4K20me1 X-enrichment in embryos compared to L3s, consistent with H4K20me1 enrichment occurring later in embryonic development [18,29]. Incomplete X-enrichment of H4K20me1 in mixed stage embryos was also reflected by less H4K20me1 ChIP-seq enrichment on the X chromosome in wild type embryos compared to L3 (Fig 7E, left panel).

The delayed H4K20me1 enrichment may also cause a delay in full dosage compensation. X chromosome expression was not fully balanced between sexes during embryogenesis (Fig 3A). While this may be due to differences in sex-biased expression between developmental stages (see discussion), it may also be due to slightly less dosage compensation due to incomplete enrichment of H4K20me1 on the X chromosomes until the comma stage of embryogenesis. Indeed, X chromosome derepression upon *dpy-27* depletion was slightly but significantly higher in L3 larvae compared to mixed stage embryos (Fig 3E, Wilcoxon rank sum test p value = 5x10^-7^). This was in spite of having more efficient DPY-27 knockdown in embryos compared to L3 larvae (S4B Fig). X-enrichment of H4K20me1 may slightly strengthen DCC mediated X chromosome repression in later development.

### In the *set-4* mutant, repression of autosomes results in a relative effect of X derepression

**Fig 8:**
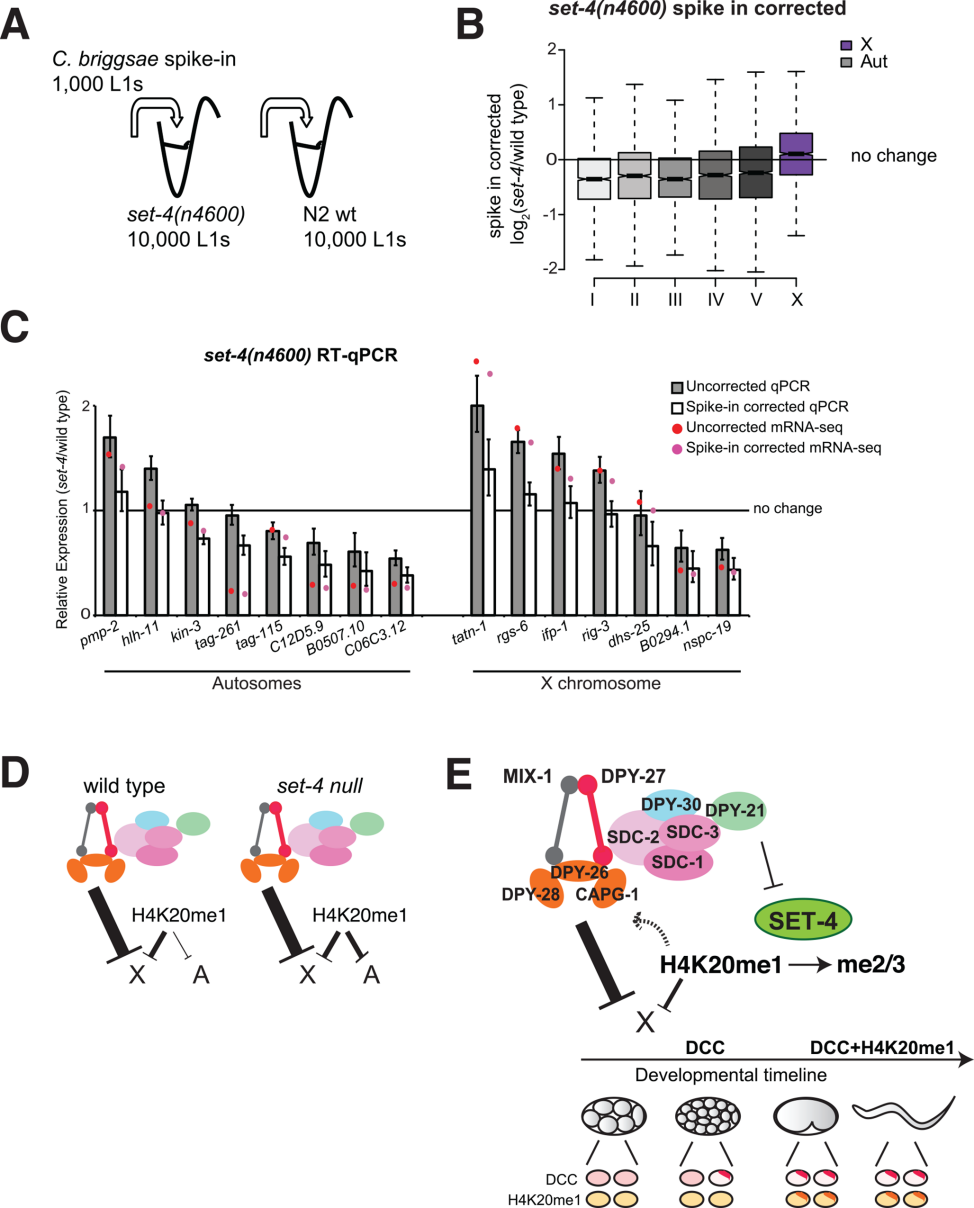
Spike-in normalized analyses of gene expression in the *set-4(n4600)* null mutant suggest that autosomal repression results in a net effect of X derepression. (A) Schematic representation of the spike-in mRNA-seq experiment. Here we counted and mixed in *C*. *briggsae* L1s with *C*. *elegans* L1s prior to total mRNA isolation. Since the spiked in *C*. *briggsae* worms are the same and mixed-in at the same proportion, we normalized expression from *C*. *elegans* to that of *C*. *briggsae*. Normalization was done by determining the coefficients of a linear regression that equalized *C*. *briggsae* expression ratio between mutant and wild type, and applying these parameters to the *C*. *elegans* expression ratios (S8B Fig). (B) Boxplot shows distribution of corrected log_2_ fold-changes in mRNA level between *set-4(n4600)* and wild type L1 larvae. (C) Comparison of differential expression values determined by spike-in mRNA-seq and RT-qPCR for a number of genes on the X and autosomes. Since RT-qPCR also relies on the assumption that the total RNA does not change between wild type and mutant, we used amplification of two *C*. *briggsae* genes to correct expression difference between wild type and mutant (see Materials and Methods). Darker bars show RT-qPCR relative expression (mutant/wild type) values and lighter bars show spike in corrected values. Circles show corresponding mRNA-seq differential expression ratios with (red) and without (pink) spike in correction. Error bars represent relative the standard error of the mean from 3 biological replicates. (D) Interpretation of the spike-in mRNA-seq and RT-qPCR data suggests that stronger repression from the autosomes in the *set-4(n4600)* mutant results in a relative increase from the X. (E) Current model for the temporal dynamics and the role of DCC and H4K20me1 in X chromosome repression. Solid arrows and their thickness indicate the level of repression. Dashed arrow represents a possible role for H4K20me1 in strengthening DCC mediated repression. Within the developmental timeline, early stage embryos before and after DCC activation, comma stage embryos, and larva are depicted. Ovals represent nuclei with DCC (top) and H4K20me1 (bottom) signals in pink and yellow. For simplicity, X chromosome localization is represented as darker colors in a single subnuclear domain. In early embryogenesis, as the DCC starts loading onto the X chromosomes, it starts repression. DCC binding leads to H4K20me1 increase on the X chromosome, which contributes slightly to repression in later development.

The small but significant X derepression observed in the *set-4(n4600)* mutant suggested that X/A balance of H4K20me1 contributes slightly to X chromosome dosage compensation (Fig 4C). To address the mechanism of this contribution, we further analyzed gene expression in the *set-4(n4600)* mutant. Since mRNA-seq measures relative expression changes, we could not distinguish if the relative X derepression seen in the *set-4(n4600)* mutant is due to repression from autosomes or an increase from the X. Therefore, we adapted the spike-in normalization scheme for mRNA-seq analysis (Fig 8A). To do this, we mixed 1,000 *C*. *briggsae* worms to 10,000 N2 (wild type) or *set-4* mutant worms before RNA extraction and mRNA-seq analysis. We used fed L1 worms where we could reliably assume that the number of cells was the same between wild type and the mutant. The replicates correlated well with each other (S8A Fig). Since spiked in *C*. *briggsae* worms were the same across all samples, we normalized *C*. *elegans* expression levels using the linear regression coefficients that equalized *C*. *briggsae* expression between wild type and mutant samples (S8B Fig).

Spike-in analysis of mRNA-seq levels in the *set-4(n4600)* mutant showed decreased expression from autosomes (Fig 8B), resulting in the relative effect of X derepression seen in Fig 4C. To validate the spike-in mRNA-seq method, we compared it to RT-qPCR analysis for a number of genes. RT-qPCR was performed on the same total RNA preparations that were used for making the mRNA-seq libraries. Since RT-qPCR also relies on the assumption that total mRNA levels are the same between the wild type and the mutant, two *C*. *briggsae* genes were used as standard. Results from standard and spike in normalized RT-qPCR results were similar to mRNA-seq analysis (Fig 8C).

### A model for the role of H4K20me1 in X chromosome dosage compensation

We reason that if H4K20me1 acts as a downstream effector of the DCC (i.e. the effect of H4K20me1 on transcriptional repression was the same on the X and autosomes), equalization of H4K20me1 between the X and autosomes should result in the same level of X chromosome derepression. Two observations suggested that while H4K20me1 is important, X/A balance of H4K20me1 is not responsible for X chromosome repression. First, upon equalization of H4K20me1 levels between X and autosomes in the *set-4(n4600)* mutant, X chromosome is only slightly derepressed (Fig 5C). Second, H4K20me1 depletion in the *set-1(tm1821)* mutant resulted in greater X derepression compared to equalization of H4K20me1 between X and autosomes in the *set-4(n4600)* mutant (Fig 5C). Thus H4K20me1 does not act as the sole downstream effector of the DCC. Based on the spike in controlled ChIP-seq and mRNA-seq analyses in the *set-4(n4600)* mutant, we propose a model that H4K20me1 enrichment only slightly represses X chromosome transcription, but H4K20me1 may positively affect DCC activity, which is primarily responsible for X repression (Fig 8E).

## Discussion

### Tolerating X chromosome dosage difference during embryogenesis

Our results indicate that in early embryogenesis, X chromosome is not fully dosage compensated in *C*. *elegans*. Then, how is the X chromosome dosage difference tolerated? First, maternal loading of mRNAs into oocytes help equalize overall X-chromosomal transcript levels between the two sexes, thus genes that are important for the development of both sexes may be provided in the oocyte. Second, X chromosome harbors fewer genes with essential functions for embryogenesis [50]. Third, dosage sensitive genes such as haploinsufficient genes are depleted from the X chromosome [51,52]. We also found that *C*. *elegans* X chromosome has fewer genes that encode for multi-subunit protein complexes, which were found to be more dosage sensitive [53–55]. There are only 16 X-chromosomal genes with a yeast homolog of a multi-protein complex [52], representing a significant depletion of such genes from the X (p< 10^−5^, Fisher’s exact test). For the remaining dosage sensitive genes on the X, in addition to DCC mediated repression, potential post-transcriptional mechanisms may act, as such regulation is extensive in *C*. *elegans* [56]. Thus, a combination of different mechanisms buffers the X chromosome dosage difference between hermaphrodite and males during early embryogenesis.

### Dosage compensation and sex-biased gene expression

Comparison of gene expression levels between hermaphrodite and male embryos reflects two genetic differences: X chromosome number and sex-biased gene expression. Others and we have shown that the X chromosome contains higher numbers of hermaphrodite-biased genes [51,57]. This may contribute to the higher X expression in hermaphrodites compared to the mixed sex embryos (Fig 3). However, most sexual dimorphism in *C*. *elegans* develops in the larval stages (reviewed in [58]), and the adult gonads contribute an especially high amount of sex-biased gene expression [51]. Indeed, mRNA-seq replicates in the hermaphrodite and mixed-sex worms clustered by developmental stage rather than sex until the young adult stage (S1A Fig). Thus, in embryos and larvae, developmental gene expression has a stronger effect on the transcriptome than sex biased gene expression. Nevertheless, there is sex-biased gene expression in embryos, including genes in the sex determination pathway (Fig 2D). Interestingly, the XSEs were dosage compensated during late embryogenesis, suggesting that the DCC acts on these genes regardless of their early hermaphrodite-biased expression [36] (S3 Fig). It is possible that the DCC mediated gene repression is chromosome-wide and superimposed onto other mechanisms of regulation that act at the level of individual genes, such as sex biased gene expression programs. Future studies that uncouple sex from X chromosome number are needed to resolve the relation between dosage compensation and sex-biased gene expression.

### Function of DCC mediated X chromosome repression

Although X chromosome dosage compensation is essential for proper development, it is still not clear how DCC mutations cause lethality (reviewed in [1]). In many animals, proper chromosome dosage is important, as trisomy or monosomy of chromosomes is not tolerated (reviewed in [59]). X chromosome dosage compensation problems in mammals and worms would cause upregulation of X-chromosomal genes. In the case of trisomies, increased expression from a set of dosage sensitive genes may cause problems, as in Down syndrome (reviewed in [60]). Additionally, increased expression from a whole chromosome pressures proteasome pathways needed to degrade the extra proteins, as was shown in yeast [61]. DCC mutations cause derepression of many X chromosomal genes that are involved in wide range of processes (Fig 6). Genes that are upregulated in the *dpy-27* mutant include several transcription factors and kinases, whose overexpression may cause off-target effects (discussed in [62]). While it is possible that the combination of a number of dosage sensitive genes on the X are responsible for the DCC phenotypes, DCC-mediated repression is applied across the X chromosome (Fig 4), suggesting that the DCC evolved to repress average X chromosomal gene dosage.

### Developmental dynamics of H4K20me1 enrichment on the X chromosomes

DCC starts binding to the X chromosomes at around the 40-cell stage, which is followed by H4K20me1 enrichment on the X, which occurs between the 100-cell and comma stages [18,29]. Since the DCC reduces SET-4 activity, and SET-4 needs previous monomethylation of H4K20 to catalyze di- and trimethylation, H4K20me1 enrichment on the X can only happen after the initial deposition of H4K20me1 during cell division. The delay in H4K20me1 enrichment during embryogenesis may be due to a requirement for cell divisions to initially deposit H4K20me1. In mammalian tissue culture cells, cell-cycle regulation of SET-1 ensures a dramatic increase in H4K20me1 on mitotic chromosomes [63,64]. H4K20me1 also increases on mitotic chromosomes in *C*. *elegans* [18,29]. Therefore, it is possible that multiple divisions are necessary to successively increase H4K20me1 on the *C*. *elegans* X chromosomes. A microscopy-based study of H4K20me1 enrichment across multiple divisions in one cell lineage can address this possibility.

### An indirect role of *set-4* in X chromosome dosage compensation

Previously, we hypothesized that the DCC increases H4K20me1 by reducing SET-4 activity on the X chromosomes [18]. Analysis of gene expression in the *set-4* null mutant suggested an effect on X chromosome dosage compensation (Fig 5C). This effect was indirect, as an average of ~20% reduction across the autosomes resulted in a relative X chromosome derepression compared to autosomes (Fig 8B). Since H4K20me1 increase in the *set-4* null mutant resulted in repression, H4K20me1 enrichment on the X chromosome may slightly contribute to repression in hermaphrodites. H4K20 methylation has a role in gene expression in other organisms as well, but the mechanisms remain unclear [65].

It is possible that H4K20me1 positively regulates chromatin compaction, thus reduces accessibility of RNA Polymerase II to the X chromosomes. H4K20me1 is enriched on mitotic chromosomes and the inactive X chromosome in mammals [65]. In *C*. *elegans*, H4K20me1 is enriched on mitotic chromosomes and across the entire X (Fig 7A), which is more compact in hermaphrodites [66]. Both *set-1* and *set-4* resulted in a relative increase in X chromosome volume compared to the autosomes in hermaphrodites [66]. While *set-4* is important for X compaction, the null mutant showed only a slight effect on X chromosome dosage compensation and does not have obvious dosage compensation phenotypes in standard laboratory conditions (Fig 5C). Therefore, X compaction by itself does not fully explain DCC mediated X chromosome repression.

Regulation of H4K20 methylation by *set-4* may be involved in additional processes. *Set-4* knockdown increased lifespan in an RNAi screen [67] and suppressed the developmental delay associated with mutant *rict-1*, a component of the target of rapamycin complex 2 [68]. Gene expression changes that occur in the *set-4* mutant may be the basis for these phenotypes. Alternatively, *set-4* may act through other non-histone substrates, as recent experiments in *D*. *melanogaster* showed that H4K20A mutation did not completely recapitulate the phenotype of the methyltransferase mutant [69].

### The role of H4K20me1 in X chromosome dosage compensation

Our working model for H4K20me1 in X chromosome dosage compensation is summarized in Fig 8E. Briefly, X-enrichment of H4K20me1 contributes slightly to X chromosome repression, but H4K20me1 by itself is not a major downstream effector of the DCC. Instead, H4K20me1 level is important for DCC-mediated repression of the X chromosomes. One possibility is that H4K20me1 may increase association of condensin DC with chromatin. In agreement with this possibility, there was slightly less DPY-27 binding in the *dpy-21(e428)* mutant embryos where H4K20me1 is no longer enriched on the X chromosome (S7C Fig). In mammalian cells, HEAT domains found in N-CAPD3 and N-CAPG2 subunits of condensin II bind to H4K20me1 containing histone tail peptides, and this interaction was proposed to increase condensin II binding on mitotic chromosomes, which are enriched for H4K20me1 [70]. The DCC contains two subunits with HEAT domains, DPY-28 and CAPG-1 [20]. Positive regulation of DCC by H4K20me1 may help maintain dosage compensation complex on the X in later developmental stages in *C*. *elegans*. Such maintenance mechanisms are involved in epigenetic gene regulation, and have been demonstrated to be important for mammalian X inactivation [71,72].

### The mechanism of transcription repression by the DCC

Previous work and our results suggest that the DCC represses X chromosome transcription. The molecular mechanism by which the DCC represses transcription remains unclear. In various organisms, condensins demonstrate activities such as positive supercoiling [73–75], single stranded DNA reannealing [76,77] and making topological links in DNA [78,79]. It is not known if condensin DC has these activities.

The DCC is enriched at active promoters [80] and the DCC reduces RNA Pol II binding to X chromosome promoters [43]. The DCC ChIP binding scores at promoters positively correlate with the amount of transcription [81]. Positive correlation between condensin binding and transcription was also seen in yeast and mammalian cells [77,82–84]. We had hypothesized that coupling the level of DCC binding to the level of transcription at each promoter could be the basis of the two-fold reduction in gene expression regardless of the expression level [80]. Indeed, GRO-seq analysis indicated that while the DCC binding positively correlates with transcription, the level of DCC mediated reduction in RNA Pol II is similar across genes transcribed at a wide range of levels [43]. Based on the observation that the DCC binds to promoters, and that yeast condensin interacts with TBP [83], it is possible that the DCC is recruited to promoters by a general transcription factor, and then halves the rate of RNA Pol II binding at each cycle of transcription.

Alternative but not exclusive DCC mechanisms have been proposed, such as compacting chromatin to reduce activators’ access to the X chromosome or changing interactions between enhancer and promoters to reduce RNA Pol II recruitment [43]. These models are based on the observations that condensins are required for chromosome condensation, and in other organisms such as *D*. *melanogaster* and mammalian cells, condensins regulate long-range chromosomal interactions [85,86]. Recently, the DCC was shown to compact the X chromosome [27], regulate sub-nuclear localization of the X [87], and form topologically associated domains on the X [88]. However, it remains unclear if these large-scale chromatin changes are the cause or consequence of increased transcription from the X chromosomes in the DCC mutants.

In mouse, *C*. *elegans* and *D*. *melanogaster*, dosage compensation complexes are targeted to and regulate the chromatin structure and transcription from the X chromosomes in one of the two sexes (reviewed in [1]). The composition of the dosage compensation complexes suggests that different complexes coopted different sets of gene regulatory mechanisms to the X chromosomes. In the case of *C*. *elegans*, duplication and divergence of a condensin subunit (*dpy-27*) created the dosage compensation specific condensin complex (condensin DC) that reduces transcription from both X chromosomes in hermaphrodites. Condensins are essential for chromosome condensation during cell division, and regulate chromatin compaction and transcription during interphase. H4K20me1 is a mitosis enriched chromatin mark that is also enriched on the dosage compensated X chromosomes in *C*. *elegans*. Our work on the DCC and H4K20me1 contributes to understanding the link between the mitotic and interphase function of condensins in chromosome condensation and gene regulation.

## Materials and Methods

### Worm strains

N2, wild type; BS553, *fog-2(oz40)*; CB428, *dpy-21(e428)V*; MK4 *dpy-27(y56)/qC1[dpy-19(e1259) glp-1(q339)] [qIs26]* III; SS1075 *set-1(tm1821)/hT2g[bli-4(e937) let-*?*(q782) qIs48] (I;III)*; MT14911 *set-4 (n4600) II*; VC199, AF16 *C*. *briggsae* wild isolate.

### Worm growth and collections

Unless otherwise noted, strains were maintained at 20°C on NGM agar plates using standard *C*. *elegans* growth methods. For staged embryo collections, worms were synchronized by bleaching gravid worms, allowing embryos to hatch overnight and growing the synchronized L1s on NGM plates for ~60 hours until the first embryos were seen in hermaphrodite gonads. Early embryos were isolated from bleached young adults, and immediately collected. Comma embryos were obtained by aging early embryos 4 hours in M9 buffer. Larvae were collected after growing hatched L1s for 6 hours (L1 larvae) or 24 hours (L3 larvae) on NGM plates. Young adults were synchronized by growing hatched L1s for 44 hours at 25°C, and collected prior to the accumulation of fertilized embryos in the gonad. Mixed stage embryos were collected by bleaching gravid adults. Developmental stages were assessed by counting DAPI stained nuclei by DAPI staining, assessing germline, male tail and vulva morphology.

Due to lethality, *set-1(tm1821)* mutants were kept heterozygous with a balancer chromosome that includes a GFP marker (SS1075 strain [18]). *set-1*/balancer heterozygous larvae were selected by picking GFP(+) animals and *set-1* homozygous mutant larvae were identified by picking GFP(-) animals. Since *dpy-27(y56)* is lethal [12,28], *dpy-27(y56)* worms were kept as heterozygotes with a balancer chromosome that encodes *rol-6(su1006)*. *dpy-27(y56)* homozygote embryos were collected by bleaching young adults without the roller phenotype. This resulted in embryos that lacked both maternal and zygotic *dpy-27*.

For DPY-27 RNAi, the bacterial strain from the Ahringer RNAi library containing *dpy-27* RNAi plasmid and control plasmid were grown and induced in 100 ml LB media for 3 hours with 0.1 mM ITPG, concentrated 10-fold and seeded onto a 10 cm NGM plate supplemented with ampicillin, tetracycline and 1mM IPTG. Synchronized L1s were placed on seeded plates and collected at 24 hours (L3 larvae) or grown to gravid adults and embryos were collected by bleaching (mixed stage embryos). At collection time, all samples were washed 3 times in M9 buffer and stored in ten volumes of Trizol (Invitrogen) at -80°C. 2–3 collections were pooled for one biological replicate.

### mRNA-seq and ChIP-seq

A summary of all the data sets and replicates are provided in S1 File. Raw data files and wiggle tracks of ChIP enrichment per base pair, and RNA-seq FPKM values are provided at Gene Expression Omnibus database (http://www.ncbi.nlm.nih.gov/geo/) under accession number [GSE67650].

For RNA preparation, samples in Trizol were frozen and thawed 3–5 times to freeze-crack worms, and phase containing the total RNA was cleaned up using the Qiagen RNeasy kit. mRNA was purified using Sera-Mag oligo(dT) beads (ThermoScientific) from 0.5–10 ug of total RNA. Stranded mRNA-seq libraries were prepared based on incorporation of dUTPs during cDNA synthesis using a previously described protocol [89]. Single-end 50-bp sequencing was performed using the Illumina HiSeq-2000. Reads were mapped to the *C*. *elegans* genome version WS220 with Tophat v1.4.1 [90] using default parameters. Gene expression was quantified using Cufflinks version 2.0.2 [91] for strand-specific reads using default parameters and supplying gene annotations. Gene expression values used were the mean of at least three biological replicates, and are provided in S2 File. Differential expression analysis was performed using DESeq2 version 1.0.17 [92] in R version 3.0.2, and the values are given in S3 File. All analyses were performed with genes that had average expression level above 1 FPKM (fragments per kilobase per million, as calculated by Cufflinks). RNA-seq data clustering was performed by K-means clustering using the Hartigan-Wong method in R version 3.02. GO term analysis was performed using GOrilla [93]. The clusters and GO analyses results are provided in S4 File.

ChIP-seq was performed as previously described [26], using H4K20me1 antibody (Abcam ab9051), a custom DPY-27 antibody [80], and AMA-1 antibody against the large subunit of RNA-Pol II (modENCODE SDQ2357). Single end reads were aligned to the *C*. *elegans* genome version WS220 using Bowtie 1.0.1 [94], allowing up to two mismatches in the seed, returning only the best alignment, and restricting a read to map to at most four locations in the genome. Peak calling and genome-wide coverage estimation was obtained with MACS version 1.4.2 [95] using mapped reads from ChIP and input. To estimate the final genome coverage, coverage per base was normalized to the genome-wide median coverage, excluding the mitochondrial chromosome, and the input was subtracted from the ChIP. Replicates were merged by averaging coverage at each base position across replicates. For peak calling, ChIP and input reads were merged from all replicates using samtools merge version 0.1.19 [96], and MACS was used for peak calling. The peak calling was also done per replicate, and peaks from the combined data that also occur in the majority of replicates were retained. For wild type and mutant comparisons, data sets were standardized by z-score transformation of the ChIP enrichment values based on the presumed background, excluding regions covering MACS peaks (e-5 cutoff).

### Analysis of expression similarity and expression distribution

The statistical framework for identifying differentially expressed genes takes into account the average and standard deviation between replicates. Genes that are not found as differentially expressed cannot be labeled as “statistically” similarly expressed, because variability between replicates may cause these genes to fail the statistical test. Therefore, to determine genes that are similarly expressed in hermaphrodite and mixed sex embryos, we constructed a 95% confidence interval around the log_2_ expression ratio for each gene. For a gene to be called similarly expressed, that confidence interval could not exceed a 30% change in expression. Distribution of similarly, equivalently and differentially expressed results are provided in S5 File.

To determine if there is two classes of DCC regulated genes on the X chromosome, we used an expectation-maximization algorithm to determine the distribution of expression changes upon *dpy-27* knockdown. A mixture distribution with two or three components was assumed and the better fit was used. Parameters of the mixture distribution were estimated using the mixtools package [97] in R version 3.0.2.

### Spike in analysis

For RNA-seq, hatched L1s were fed on OP50 seeded plates for 6 hours, collected, counted three times and diluted to the desired number of worms based on the average count. 10,000 *C*. *elegans* L1s were mixed in with 1,000 *C*. *briggsae* L1s. RNA-seq was performed as described above. mRNA-seq reads were mapped to the *C*. *elegans* genome version WS220 and *C*. *briggsae* genome version WS224 using TopHat (Trapnell et al 2010) version 2.0.11 and reads mapping to both genomes were discarded from further analyses. Raw counts for each gene were estimated with htseq-count version 0.6.1 [98]. All read counts were normalized based on total reads and the median across three replicates was determined. A linear model was fitted to the highly expressed genes from wild type and mutant *C*. *briggsae* spike-in data, whose coefficients were used to correct the *C*. *elegans* expression data.

For ChIP-seq, 0.1–0.2 mg of ChIP-extract prepared from *C*. *briggsae* embryos were added to 1–2 mg *C*. *elegans* ChIP extract. ChIP and corresponding input reads were mapped to the *C*. *elegans* genome version WS220 and *C*. *briggsae* genome version WS224 using Bowtie version 1.0.1 [94] and reads mapping to both genomes were discarded. To determine a spike-in normalization factor, mapped reads were first normalized to total reads and then the ratio between ChIP and input was calculated for uniquely mapped reads to *C*. *elegans* or *C*. *briggsae*. The ratio of *C*. *elegans* and *C*. *briggsae* ChIP/Input between the mutant and wild type was used to correct the genome coverage in the mutant.

### RT-qPCR analysis

We used the total RNA that was isolated for the spike in libraries. 20 nanograms of total RNA was used per 20 microliter one-step RT-qPCR reaction (KAPA Biosystems). To eliminate cross amplification between *C*. *elegans* and the *C*. *briggsae* spike in RNA, primers were designed to amplify *C*. *elegans* that do not amplify in the *C*. *briggsae* genome. *C*. *briggsae* genes *CBG27711* and *CBG25839* were selected as the spike in normalization gene based on L1 expression, lack of *C*. *elegans* homolog and lack of amplification from the *C*. *elegans* template cDNA. No-RT and opposite species template RNA reactions were used as controls to confirm specificity of primers. Relative fold change between *set-4(n4600)* and wild type was calculated using the ΔC_t_ method and spike in normalized changes were calculated using the ΔΔC_t_ method using the averageΔC_t_ of *C*. *briggsae* genes *CBG27711* and *CBG25839* between wild type and *set-4* as the second normalization parameter [99]. Primer sequences are as follows: *kin-3* forward TTTCAAGGGACCCGAGCTTC, *kin-3* reverse GTGTCGCCCGAGAATATCGT, *pmp-2* forward CCTGGAGTGGTTGGAATGCT, *pmp-2* reverse TCTTGCCAGTTTGCCCAGAA, *hlh-11* forward TTCGTTCGGATAGCGCAGAA, *hlh-11* reverse GGTGCCATTCGTGCATTTGT, *tag-115* forward GTTCAGGACCCCGGTCAAAA, *tag-115* reverse TTGGAAGTGATCGCTGACCG, *rig-3* forward GCACCAGGAAACAAGCAAGG, *rig-3* reverse TTGCATCACGGACGTGGTTA, *ifp-1* forward TCCAGAGCGACAGTAGTGGA, *ifp-1* reverse TAGTAGTTCGTGCCGGCTTC, *rgs-6* forward AATGTGAGCGAACCCAGCAA, *rgs-6* reverse CGCCTCGTCGCCTATTTTTG, *dhs-25* forward CTGGGAGGACGAATTGCTGT,*dhs-25* reverse CCCCTTCACACTGTCTGCAT, *CBG27711* forward ATGAGCATAATTCAGAGAGAAAATTTT, *CBG27711* reverse CTAGGCATCTATAATTAGGTT, *tag-261* forward CGTAGATGCACGTCTTCAAGC, *tag-261* reverse TCTCCGCCTCTTTGAGACCTC, *C12D5*.*09* forward TGACTTTTTCTCGACGGATTTG, *C12D5*.*09* reverse CACACCTTCTTTCGTTGGCG, *C06C3*.*12 f*orward TCGGTGGACCGATTCTAATGAA, *C06C3*.*12* reverse TTAGCCCACTTCGACTGAGG, *B0507*.*10* forward TCTCAGGAATTGCTAATTCGGC, *B0507*.*10* reverse GGCTCTGTGGCGGTTGATAA, tatn-1 forward CGCTTCTTGAGCAAGCCAAA, tatn-1 reverse CTGCGATCTCACTTGGTGGA, *nspc-19* forward CATCCTTGCTGCTCTCTGCT, *nspc-19* reverse TTCTCCACCGTTGACACCTG, *B0294*.*1* forward ATCCGCAACCAACTCTCCTG, *B0294*.1 reverse CAAGGTTTTCTCCATTCTTTGGCA, *CBG25839* forward CGCGTCTAAACCAGCCAAAC, *CBG25839* reverse AGCTGAGCGAGATGAACACC.

## Supporting Information

S1 Fig Correlation of mRNA-seq data sets. (A) Pair-wise spearman rank correlation coefficients between each RNA-seq data set were calculated by the gene read counts and were hierarchically clustered for hermaphrodite and mixed sex mRNA-seq replicates at different developmental stages. (B) Same as in (A) using replicates of wild type and mutant or RNAi data sets.
(EPS)

S2 Fig Collection and expression in early embryos. (A) Representative images from early embryo collections show embryos that have been fixed with methanol and stained with DAPI. At the bottom, distribution of number of nuclei in hermaphrodite (left panel) and mixed sex (right panel) early embryo collections is shown. (B) Histograms show distribution of log_2_ fold-differences between hermaphrodite and mixed sex early embryos on the X chromosome (left panel) or autosomes (right panel). Differentially expressed genes (DESeq2, padj <0.05) are shown in dark grey.
(EPS)

S3 Fig X and autosomal signal element expression. (A) Expression values (FPKM) for each of the known and mapped XSE and ASEs are shown in the hermaphrodite and mixed sex mRNA-seq data sets. Error bars represent relative the standard error of the mean from the biological replicates. (B) The expression values from our embryo data agree with data published in hermaphrodite embryos with greater time resolution [30]. Two representative genes are shown.
(EPS)

S4 Fig Analysis of *dpy-27* RNAi and *dpy-21* mutant worms. (A) Representative distribution of stages for mixed stage embryos isolated from gravid adults by bleaching. (B) Reduction of DPY-27 protein levels upon *dpy-27* RNAi. Western blot shows DPY-27 protein levels in RNAi and control vector collections. Knockdown percentage was calculated by comparing DPY-27 signal in RNAi versus vector control using tubulin as the negative control. (C) mRNA-seq log_2_ fold change between mutant and wild type early embryos were clustered using k-means clustering. In early embryos, transcriptional effects of *dpy-27(y56)* and *dpy-21(e428)* were more distinct on the X chromosomes compared to mixed stage embryos and L3 larvae.
(EPS)

S5 Fig Sex-ratio and the effect of *dpy-27* and *dpy-21* on newly expressed gene clusters for genes that are expressed highly in both sexes. Hermaphrodite expression values for consecutive developmental time points were clustered and clusters of genes upregulated in the older point were identified and marked with asterisk. Newly and highly expressed genes were identified by taking those that were not expressed in the earlier time point (FPKM<1), and highly expressed in the consecutive time point (FPKM>10). Hermaphrodite/ mixed sex log_2_ expression ratios, log_2_ fold changes upon *dpy-27* (*y56* or RNAi) and *dpy-21(e428)* are shown for the X and autosomes. For comparison, ratios for all highly expressed genes are also shown.
(EPS)

S6 Fig Correlation between ChIP-seq data sets. (A) Pair-wise spearman rank correlation coefficients between each ChIP-seq data replicates were calculated from average ChIP-seq enrichment within 1-kb windows tiled across the genome. The correlations were hierarchically clustered and represented as a heat map. (B) Same for the spike in ChIP-seq data using uniquely mapped *C*. *elegans* reads.
(EPS)

S7 Fig H4K20me1 ChIP-seq analysis with respect to gene expression in mixed embryos. (A) H4K20me1 ChIP-seq enrichment scores were plotted across transcription start sites (TSS) in wild type and *dpy-21* and *set-4* mutant L3s (top 3 plots) and mixed embryos (bottom 3 plots). Data were averaged for expressed (top 20% by mRNA-seq in N2) and silent (bottom 20%) genes on the X chromosome (red) and autosomes (blue). (B) Spike in corrected H4K20me1 enrichment by chromosome, same as Fig 7C, but for mixed embryo data set. Model shows the accumulation of H4K20me1 in wild type and *set-4(n4600)* mutants in mixed embryos and larvae. (C) DPY-27 ChIP enrichment averaged across all Wormbase annotated transcription start sites (WS220) or newly annotated GRO-seq defined start site [43]. DPY-27 binding was slightly reduced in *dpy-21(e428)* and not changed in *set-4(n4600)* mutants.
(EPS)

S8 Fig Correlation and normalization of mRNA-seq spike in data sets. (A) Pair-wise spearman rank correlation coefficients between mRNA-seq expression values calculated from uniquely mapping *C*. *elegans* and *C*. *briggsae* reads. (B) Top left plot shows the correlation of gene expression for the highly expressed spiked in *C*. *briggsae* genes, as median read counts from the three replicates. The linear regression coefficients from the fit (red line) were corrected to match equal expression (grey line) and the corrected values were plotted in the top right panel. Bottom left panel shows gene expression for *C*. *elegans* genes in wild type and mutant before correction. Bottom right panel shows the gene expression after correction with the linear regression coefficients calculated for the *C*. *briggsae* spike-in expression.
(EPS)

S1 File The list of mRNA-seq and ChIP-seq data sets and their GEO accession numbers. (XLSX)

S2 File Average FPKM expression levels for mRNA-seq data sets. (XLSX)

S3 File DESeq2 analysis results for differential expression. (XLSX)

S4 File Gene clusters from various comparisons and GO Term analyses used for Fig 6. (XLSX)

S5 File Numbers of similarly and differentially expressed genes between sexes, and genes repressed by the DCC. (XLSX)
