# Correction: Developmental Dynamics of X-Chromosome Dosage Compensation by the DCC and H4K20me1 in *C*. *elegans*

**DOI:** 10.1371/journal.pgen.1005899

**Published:** 2016-02-22

**Authors:** Maxwell Kramer, Anna-Lena Kranz, Amanda Su, Lara H. Winterkorn, Sarah Elizabeth Albritton, Sevinc Ercan

There are errors in the second and third sentences from the end of the Abstract. These sentences should read “H4K20me1 depletion in the *set-1* mutant showed greater X derepression compared to equalization of H4K20me1 levels between X and autosomes in the *set-4* mutant, indicating that H4K20me1 level is important, but X to autosomal balance of H4K20me1 contributes slightly to X-repression. Thus H4K20me1 is not only a downstream effector of the DCC.”

The last sentence of the Introduction is incorrect. The sentence should read “Our results suggest that H4K20me1 levels are important for X chromosome dosage compensation, but H4K20me1 does not act only as a downstream effector of the DCC.”
